# DNA Methylation in Nasal Epithelium: Strengths and Limitations of an Emergent Biomarker for Childhood Asthma

**DOI:** 10.3389/fped.2020.00256

**Published:** 2020-05-15

**Authors:** Giulia Solazzo, Giuliana Ferrante, Stefania La Grutta

**Affiliations:** ^1^Institute for Research and Biomedical Innovation (IRIB), National Research Council (CNR), Palermo, Italy; ^2^Department of Health Promotion Sciences, Maternal and Infant Care, Internal Medicine and Medical Specialities, University of Palermo, Palermo, Italy

**Keywords:** DNA methylation, nasal epithelium, asthma, biomarker, children

## Abstract

Asthma is one of the most widespread chronic respiratory conditions. This disease primarily develops in childhood and is influenced by different factors, mainly genetics and environmental factors. DNA methylation is an epigenetic mechanism which may represent a bridge between these two factors, providing a tool to comprehend the interaction between genetics and environment. Most epidemiological studies in this field have been conducted using blood samples, although DNA methylation marks in blood may not be reliable for drawing exhaustive conclusions about DNA methylation in the airways. Because of the role of nasal epithelium in asthma and the tissue specificity of DNA methylation, studying the relationship between DNA methylation and childhood asthma might reveal crucial information about this widespread respiratory disease. The purpose of this review is to describe current findings in this field of research. We will present a viewpoint of selected studies, consider strengths and limitations, and propose future research in this area.

## Background

Asthma is a chronic inflammatory condition that affects the airways and is defined by recurrent episodes of wheeze, breathlessness, chest tightness and/or cough associated with variable expiratory airflow limitation (1). Despite environmental exposures explaining about one third of risk factors for asthma, genetic factors are also responsible for vulnerability to the disease (1–3). Epigenetic marks have been suggested as a link between genetic predisposition and environmental exposure, providing a special chance to clarify the relationship between genetics and environment (4, 5).

Much evidence suggest that epigenetic processes, such as DNA methylation, play a role in asthma susceptibility (6). Although most of epidemiological studies have been conducted using blood samples, there is evidence that the study of the nasal methylome allows for making more reliable conclusions about DNA methylation in the lungs. Recently, Brugha et al. provided evidence that the bronchial epithelium and blood are twice as distant as the bronchial and nasal epithelium, emphasizing that DNA methylation in blood samples may not be informative enough to draw conclusions about methylation marks in the airways (7). Thanks to the tissue specificity, the DNA methylation of nasal epithelium may be crucial for understanding the complex molecular patterns involved in asthma. Currently, few studies considered nasal epithelium in pediatric asthma showing that the nose is both a remarkable and less-invasive surrogate for the bronchi in transcriptional profiling studies (8) and discriminates between stable and acute asthma in children (9).

One of the first studies in this field was performed by Baccarelli et al. in 2012 (10). Using pyrosequencing, the authors analyzed the DNA methylation of interleukin-6 (*IL-6*) and nitric oxide synthase (*iNOS*) promoters, and DNA methylation of repetitive elements: Alu (*Arthrobacter luteus*) and LINE-1 (Long Interspersed Nuclear Elements). This revealed that asthmatic children who had higher values of fractional exhaled nitric oxide (FeNO) showed a hypomethylation of the *IL-16* and *iNOS* promoters. More recently, few studies performed epigenome-wide association analysis between the nasal methylome and pediatric asthma (11–14).

In this review, we aim to summarize current evidence about the relationship between DNA methylation in nasal epithelium and childhood asthma. Through this, we will present a viewpoint of selected studies considering the current strengths and limitations and introducing a future scenario in this research field.

## Epigenetics Mechanism: The Basics

The term epigenetics refers to DNA modifications that do not change the gene sequence. Modifications in DNA methylation, histone proteins that are responsible for DNA packaging, and microRNAs patterns can change genome function under different environmental stimuli (15). DNA methylation is a well-known epigenetic marker. This modification occurs in the cytosine residue of cytosine-phosphate-guanine (CpG) dinucleotides. Approximately 80% of all CpGs in the mammalian genome are estimated to be methylated. The remaining CpG residues, referred to as CpG islands, are predominantly situated in promoters of genes that are constitutively or inducible active. Usually these promoters are >500 base pairs long and more than 55% of each promoter consist of GC (16, 17). The methylation of these sites is produced by specific enzymes, the DNA methyltransferases (DNMTs). These enzymes are involved in several mechanisms such as transcriptional regulation, genomic stability, chromatin structure modulation, X chromosome inactivation, and the silencing of DNA transposable elements (6).

## Nasal Epithelium Sampling

The nasal brushing technique is the most commonly used in children for collecting nasal epithelium samples (10–14). This method has been widely used to study chronic airway diseases including cystic fibrosis, asthma, and other obstructive respiratory diseases. Cells in nasal epithelium have properties resembling bronchial epithelial cells and nasal brushing is much less invasive than bronchial brushing or bronchoalveolar lavage; thus, this technique represents a good surrogate model for lower respiratory tract studies (18). Usually, cells in the nasal epithelium are collected by brushing the inferior turbinate with a cytobrush, but it is also possible to collect nasal cells from the anterior nares by brushing the nasal bone and vigorously scrubbing the nares (19).

## Evidence Of The Role Of DNA Methylation In Nasal Epithelium On Children With Asthma

To locate evidence for the review topic, we conducted a keyword search through the PubMed database including the following criteria: (1) biological sample: nasal epithelium; (2) study population age: between 1 month and 20 years; (3) study area: metropolitan; (4) outcomes of interest: atopic and non-atopic asthma; (6) biological modification investigated: DNA methylation; (7) type of analysis: epigenome-wide or pyrosequencing analysis; (8) studies published from 2012 to 2019, as this range includes the first study published on this topic and the most recent studies; (9) language: English. We used the following search strategy: children [ALL] AND DNA methylation [ALL] AND nasal epithelium [ALL]; nasal epithelium. Abstracts (i.e., conference proceedings) and reviews were excluded. Among the 15 studies identified, eleven were excluded due to the following reasons: two studies used nasal cells to replicate results from other tissues, so that nasal epithelium was not the main tissue investigated (19, 20); one study analyzed DNA methylation in relation to single-nucleotide polymorphisms (SNPs) and gene expression in childhood asthma (21); seven studies did not focus the association between DNA methylation and asthma, but other topics such as lung function and corticosteroid treatment response (7, 22–27); one study was conducted on newborns and findings were verified in another cohort of children to characterize early DNA methylation patterns in airways depending on gender (28). Finally, 4 studies were included in this review (Table 1).

**Table 1:** Main characteristics of the four studies selected.

**References**	**Title of manuscript**	**Cohort**	**Age**	**Methods**	**Main Findings**
Yang et al. (11)	The nasal methylome and childhood atopic asthma	36 children with persistent atopic asthma 36 control subjects *1*st* validation* 30 asthmatic children 36 control subjects *2*nd* validation* 12 adults with atopic asthma 12 control subjects	10-12 years	Illumina Infinium Human Methylation450 BeadChips	119 DMRs^a^ associated with 118 unique genes and 118 DMPs^b^ associated with 107 unique genes.
Zhang et al. (12)	Nasal DNA methylation is associated with childhood asthma	29 sibling pairs discordant for asthma 54 sibling pairs for verification	Asthmatics 12.01 years No-Asthmatics 11.35 years	Illumina Infinium Human Methylation450 BeadChips	Three CpGs^c^ associated with asthma (cg00112952, cg14830002, cg23602092).
Forno et al. (13)	DNA methylation in nasal epithelium, atopy, and atopic asthma in children: genome-wide study	483 Puerto Rican children 72 African American children 432 European children	9-20 years	Illumina Infinium Human Methylation450 BeadChips	30 top CpGs^c^ in or near genes implicated in epithelial barrier function and in other genes involved in airway epithelial integrity and immunity
Cardenas et al. (14)	The nasal methylome as biomarker of asthma and airway inflammation in children	547 children	12.9 years	Infinium MethylationEPIC BeadChip	20 top differentially methylated CpGs^c^ associated with current asthma

a *Differentially methylated region (DMR)*.

b *Differentially methylated probe (DMP)*.

c *Citosine-phosphate-guanine (CpGs)*.

In 2017, Yang et al. (11) compared DNA methylation markers in genome and gene expression in 36 children with persistent atopic asthma and 36 non-asthmatic children. The results from this analysis were verified using a child' population of 30 subjects (aged 10 to 12 years) with atopic asthma vs. the same control group, as shared control samples [*N* = 36], and validated in an independent adult population (ranged 27 to 74 years) with atopic asthma [*N* = 12] and without asthma [*N* = 12]. The authors identified 119 epigenome-wide significant differentially methylated regions (DMRs) associated with 118 unique genes and 118 differentially methylated probes (DMPs) associated with 107 unique genes. Among 186 allergic asthma-associated differentially methylated genes (DMR and/or DMP), they found genes with an established role in asthma and atopy, immunity, in addition to genes related to extracellular matrix, cell adhesion, epigenetic regulation, and airflow obstruction. Gene expression analysis identified 53 differentially expressed genes. Among those genes, 32 had significant methylation-expression relationships within 5 kb. This was the first epigenome-wide association study on childhood asthma. Advantages of this type of analysis are that it detects DNA methylation signals in different gene regions and eventually allows to study how the methylation status of these gene regions relates to their expression. This study detected new genes with an expression status that can be involved in childhood asthma.

In 2018, Zhang et al. (12) used a cohort of 29 sibling pairs discordant for asthma. This epigenome-wide analysis identified six CpG sites for which methylation was nominally associated with asthma status. Four of them were placed in promoter regions and three of these sites were verified in a cohort of 54 sibling pairs through pyrosequencing. Two CpG sites (cg00112952 and cg14830002) were identified within the promoter region of *OR2B11*(olfactory receptor family 2 subfamily B member 11), and the other was located in the *TET1* (tet methylcytosine dioxygenase 1) promoter (cg23602092), which was already identified in a previous study on children (26). The *OR2B11* is a gene located in chromosome 1 which encodes an olfactory receptor that interacts with odorant molecules in the nose to initiate a neuronal response. The protein encoded by *TET1* is a demethylase that belongs to the TET (ten-eleven translocation) family. Interestingly, a previous study in humans (26) proposed a role for *TET1* in the pathogenesis of pediatric asthma. The finding that cg14830002 is associated with allergies in non-asthmatics and that cg23602092 is associated with asthma symptoms reveals new disease-contributing epigenetic mechanisms and novel CpG sites in nasal epithelial cells which could serve as useful asthma biomarkers.

In 2019, Forno et al. (13) carried out a genome-wide study of DNA methylation in nasal epithelium and atopy or atopic asthma in 483 Puerto Rican children and young adults (9-20 years). The authors identified specific methylation profiles associated with atopy or atopic asthma. In particular, 30 top CpG sites were selected. Many genes reported in this study are involved in epithelial barrier mechanisms and immunity, like *CDH26* (cadherin 26), *CDHR3* (cadherin related family member 3), *GJA4* (gap junction protein alpha 4), and *CAPN14* (calpain 14). Among genes of the 30 top CpG sites, there were also genes involved in atopy and inflammation like *NTRK1* (neurotrophic receptor tyrosine kinase 1), and lung function like *SLC9A3* (solute carrier family 9 member A3). The authors detected a hypomethylation and increased expression of *NTRK1* in atopy; this gene is induced by interleukin 13 (IL-13) and is increased in eosinophilic esophagitis. *SLC9A3* gene codes for an Na^+^/H^+^ exchanger and has been linked to reduced lung function in cystic fibrosis. Furthermore, the authors described two genes, which are expressed in many tissues, including the lung: *PCSK6* (proprotein convertase subtilisin/kexin type 6) encodes a protease constitutively secreted in the extracellular matrix, while the product of *METTL1* (methyltransferase like 1) gene contains a conserved S-adenosylmethionine-binding motif and is inactivated by phosphorylation. Notably, the 30-CpG panel was able to discriminate atopic subjects in three cohorts regardless of racial and ethnic differences.

In the same year Cardenas et al. (14) performed an epigenome-wide association study (EWAS) in children with asthma (mean age 12.9 years) and healthy controls. Using nasal swabs, the authors reported the top 20 differentially methylated CpGs associated with current asthma compared to non-asthmatic subjects. They also reported the differential DNA methylation of genomic regions of three genes previously associated with asthma: *TNIP-1* (TNFAIP3 interacting protein 1), *IL-13*, and *CHI3L1* (chitinase 3 like 1) (29–31), showing that the nasal methylome may serve as a reliable biomarker of asthma in children.

According to the findings described in the aforementioned four studies, a few loci do overlap. In the 30 top CpGs found by Forno et al. (13), *GJA4* and *METTL1* sites overlap with CpGs previously described in the study by Yang et al. (11), which had shown a hypomethylation in these sites in asthma cases when compared to controls. Interestingly, Forno et al. (13) replicated their top results in the Yang and colleague's cohort and 28 of 30 CpGs were significant (*p*-value < 0.01). Among the results described (11, 13), in both studies *PCSK6* was hypomethylated in children with atopy or atopic asthma compared to controls. Cardenas et al. (14) found two CpGs, cg22855021 in *TSHR* (thyroid stimulating hormone receptor) and cg24707200 in *NTRK1*, associated with allergic asthma and also overlapping the top results described in the study by Forno et al. (13). The authors replicated their top results in Forno et al. (13) and Yang et al. (11) cohorts, finding some genes which appeared significant in both the cohorts. In particular, they observed significant sites in *EPX* (eosinophil peroxidase) and *EVL* (Enah/Vasp-like) genes for asthma.

The top five results of each study selected in the current review are described in Table 2. Overall, all these studies identified two main groups of loci: those in genes related to immunity, like *ALOX15* (arachidonate 15-lipoxygenase), and those in genes involved in epithelial cells function and integrity like receptor, extracellular matrix protein and connexin as *GJA4, OR2B11, NTRK1*. These findings proved the suitability of using the nasal methylome in research settings and pave the way toward developing a nasal methylation panel for future clinical applications in childhood asthma.

**Table 2:** Top five results of the four studies selected.

**Gene**	**Official Full Name**	**CpG^a^ or DMR^b^**	***p*-value**	**Chromosome**	**References**
*ALOX15*	Arachidonate 15-lipoxygenase	4541333-4541334	7.82 x 10^−4^	chr 17	Yang et al. (11)
*HLA-DPA1*	Major histocompatibility complex, class II, DP alpha 1	33041220-33041697	1.04 x 10 ^−6^	chr 6	Yang et al. (11)
*GJA4*	Gap junction protein alpha 4	35258778-35258933	6.00 x 10 ^−3^	chr 1	Yang et al. (11)
*POSTN*	Periostin	38172802-38172803	7.25 x 10 ^−4^	chr 13	Yang et al. (11)
*LDLRAD3*	Low density lipoprotein receptor class A domain containing 3	36030085-36030086	6.23 x 10 ^−6^	chr 11	Yang et al. (11)
*OR2B11*	Olfactory receptor family 2 subfamily B member 11	cg00112952	0.004	chr 1	Zhang et al. (12)
*OR2B11*	Olfactory receptor family 2 subfamily B member 11	cg14830002	0.001	chr 1	Zhang et al. (12)
*TET1*	Tet methylcytosine dioxygenase 1	cg23602092	0.016	chr 10	Zhang et al. (12)
*ATP9B*	Atpase phospholipid transporting 9B	cg26017880	0.018	chr 18	Zhang et al. (12)
*LAMA5*	Laminin subunit alpha 5	cg14007090	0.023	chr 20	Zhang et al. (12)
*METTL1*	Methyltransferase like 1	cg20372759	2.18 × 10^−22^	chr 12	Forno et al. (13)
*GJA4*	Gap junction protein alpha 4	cg15006973	8.53 × 10^−21^	chr 1	Forno et al. (13)
*NTRK1*	Neurotrophic receptor tyrosine kinase 1	cg24707200	8.53 × 10^−21^	chr 1	Forno et al. (13)
*PDE6A*	Phosphodiesterase 6A	cg08844313	8.53 × 10^−21^	chr 5	Forno et al. (13)
*FBXL7*	F-box and leucine rich repeat protein 7	cg00664723	8.53 × 10^−21^	chr 5	Forno et al. (13)
*NOS1AP*	Nitric oxide synthase 1 adaptor protein	cg04165922	0.013	chr 1	Cardenas et al. (14)
*COPS8*	COP9 signalosome subunit 8	cg10117579	0.013	chr 2	Cardenas et al. (14)
*GLB1*	Galactosidase beta 1	cg24113459	0.013	chr 3	Cardenas et al. (14)
*KCNH2*	Potassium voltage-gated channel subfamily H member 2	cg22060869	0.013	chr 7	Cardenas et al. (14)
*MSI2*	Musashi RNA binding protein 2	cg04727951	0.013	chr 17	Cardenas et al. (14)

*The genes highlighted with a light gray background are involved in immunity, the genes highlighted with a dark gray background encode for extracellular matrix, receptor and other membrane proteins. The genes with a white background are involved in other functions*.

a *Citosine-phosphate-guanine (CpGs)*.

b *Differentially methylated region (DMR)*.

## Comparison of Selected Studies

All the studies selected in the current review were focused on asthma and atopic asthma (11–14). In all the studies asthma was defined by a medical diagnosis. In particular, Yang et al. (11) defined asthma following these criteria: a physician diagnosis and positive skin prick-test to at least one indoor aeroallergen. Zhang et al. (12) used a diagnosis of asthma acquired from parental reports and confirmed by medical records. Forno et al. (13) described atopic asthma as a doctor diagnosis plus at least one episode of wheeze in the last 12 months and at least one positive IgE to common aeroallergens. Finally, in the study by Cardenas et al. (14), asthma was obtained from parental report of a medical diagnosis plus report of wheeze or asthma medication in the past year and any positive IgE to common indoor or outdoor aeroallergens. Only Zhang et al. (12) reported the level of asthma severity, defined by symptom frequency using a respiratory symptom score.

With regard to study population, three studies (11, 12, 14) were performed in cohorts aged around 12 years, while Forno et al. (13) used a cohort of subjects aged between 9 and 20 years. The participants' ethnicities also differ between the studies. In Yang et al. (11), most of the participants were African Americans and the rest were Hispanic or Latino; the Zhang et al. (12) cohort included 96% African Americans; Forno et al. (13) recruited Puerto Rican children (all Hispanic or Latino); in the study by Cardenas et al. (14), 67.1% were white and only 16.1% were black. Since DNA methylation patterns can be influenced by age and ethnicity, using cohorts with a small age range and with a homogenous race may limit the generalizability of findings. To overcome this issue, the results by Yang et al. (11) were validated in two different cohorts, one of which was a group of adult Caucasians. To confirm the association between asthma and the six CpGs found, Zhang and colleagues (12) replicated their results using data from the Genomic of Secondhand smoke Exposure in Pediatric (GSEP) study and the Public Inner-City Asthma Consortium (ICAC) study. The top 30 results by Forno et al. (13) were replicated in two cohorts: the Yang et al. (11) cohort and the Prevention and Incidence of Asthma and Mite Allergy (PIAMA) cohort (32). Finally, also Cardenas et al. replicated their results using data from Yang et al. (11) and Forno et al. (13).

With regard to nasal sampling, three of the selected studies used cells from the inferior turbinate (11–13), while Cardenas et al. (14) performed their analysis on nasal cells from the anterior nares. The latter technique is easier to perform, and it does not necessitate a speculum to visualize nasal anatomy or specialized training. One possible limitation could be the lower average of respiratory epithelial cells collected (65%) compared to the inferior turbinate (99%). Nonetheless, the patients' discomfort is lower for anterior nares compared to inferior turbinate sampling (33). This is particularly important in pediatric settings where collecting samples by brushing the anterior nares would be an easier and less invasive method for nasal sampling.

The epigenome-wide DNA methylation was measured on Illumina's Infinium Human Methylation 450k BeadChip in three studies (11–13), while Cardenas et al. (14) performed the measurements with the Infinium MethylationEPIC BeadChip. The difference between these two arrays is that the Infinium Human Methylation 450 k BeadChip measures methylation at 450,000 CpG sites in the genome, while Infinium MethylationEPIC BeadChip measures methylation at more than 850,000 CpG sites. However, they both use the same technology.

The statistical analysis process was similar among the selected studies. The authors identified DMRs and they adjusted for false discovery rates (FDR). However, Yang et al. (11) also analyzed the gene expression and investigated the relationship between DMRs and gene expression changes. Zhang et al. (12) validated the association analysis between DNA methylation and asthma in candidate sites using pyrosequencing. Forno et al. (13) determined if significant methylation signals were associated with gene expression with a transcriptome-wide analysis. Cardenas et al. (14) also calculated the DNA methylation age using an online calculator (https://dnamage.genetics.ucla.edu/). A further strength in the studies performed by Forno et al. (13) and Cardenas et al. (14) is the large sample size, which increased the statistical power. However, to consider the effect size, Zhang and his colleagues (12) performed a sensitivity analysis. Finally, whereas three of the selected studies evaluated the impact of environmental exposures using questionnaires (11, 13, 14), the study by Zhang et al. (12) used a cohort of sibling-pairs, which allowed a better control of environmental confounding factors that may modify DNA methylation and contribute to asthma. Notably, in all the studies, analyses were adjusted for smoke exposure, which is known to affect DNA methylation (33).

## Future Perspectives

Previous studies investigating DNA methylation in blood samples have detected loci which have been found in the nasal epithelium. In the study by Forno et al. (13), *PCSK6* and *METTL1* were top results, and they had been previously reported in blood samples in a case-control study performed in asthmatic children and healthy subjects (34). Also Cardenas et al. (14) reported genes that were previously discovered in the same study (34), among which a specific DNA methylation mark, the *RUNX3* (RUNX family transcription factor 3), which is involved in T cell maturation. Findings from epigenome-wide meta-analysis (20) also suggested that CpG sites involved in inflammation are associated with asthma in both blood and nasal epithelium. Accordingly, methylome marks in asthma appear to be stable in both blood and nasal cells. The relationship between these two biosamples is still not clear. However, the finding of new genes in the studies included in the current review suggests that DNA methylation in the nasal epithelium may provide more information about airway epithelium pathways involved in childhood asthma. For instance, a study in nasal epithelium found for the first time a different methylation status in one gene, *SLC9A3* (solute carrier family 9 member A3), encoding for an Na+/H+ exchanger which was associated with decreased in lung function in cystic fibrosis (13). According to the available evidence, it seems that the methylome changes in blood samples often involved inflammatory mediators, while the methylome changes in nasal cells also included many proteins of extracellular matrix and membrane proteins. In particular, the pathway in nasal cells of asthmatic subjects of genes involved in extracellular matrix organization and disassembly, collagen catabolic and metabolic processes, suggests an important role of dysfunctional airway epithelium in asthma. However, it is necessary to add more epigenome-wide association analysis and to compare the results from studies in both the biosamples, in order to clarify how the information from blood and nasal epithelium differ, and then optimize their use as biomarkers.

With respect to study design, further studies in longitudinal cohorts are required to develop predictive nasal methylation panels suitable for early prediction of asthma, as well as gene candidate studies to analyze genes found to be significantly related with asthma. Since sensitization to aeroallergens has been found to drive an overlap between nasal methylome correlated with asthma and rhinitis (35), future studies should carefully consider the coexistence of rhinitis when investigating the association of DNA methylation with asthma in nasal epithelium. Furthermore, it would be fascinating to study the influence of aging on DNA methylation on this tissue, which is particularly exposed to environmental factors. Indeed, exploring how the estimated epigenetic age differs across a population of the same age may contribute to understand the effect of endogenous or exogenous stress factors on aging (36). Epigenetics biomarkers of aging, otherwise known as “the epigenetic clock,” have already been studied widely in aging-associated diseases, but evidence about the epigenetics clock's role in pediatric allergy and asthma is scant (36). To our knowledge, only one study used the nasal methylome to estimate epigenetic age acceleration in children (14), reporting a significant epigenetic age acceleration in subjects with current asthma and a greater age acceleration for those with allergic asthma. Since several age-related changes occur in lung physiology and morphology and likely have an impact on asthma, introducing aging information will allow the use of DNA methylation as a reliable biomarker for asthma in children, and might contribute to the development of antiaging therapies.

## Conclusions

Studies in children suggest that differences in DNA methylation are associated with asthma. Emerging findings in this field of research suggest that the nasal epithelium can be used to reliably infer the presence of asthma. In particular, recent epigenome-wide association studies detected new differentially methylated genes involved in solute carriers and membrane transport proteins and confirmed the association with genes involved in the immune system for asthma. However, there is limited overlap between the studies which prohibits firm conclusions from being draw on the data. This could be due to different study design and heterogeneous techniques as well as to the very limited number of studies in this field. Therefore, extending this analysis using longitudinal cohorts and analyzing gene candidates selected from relevant genes could be a promising evolution of this research area.

## Author Contributions

GS, GF, and SL provided substantial contributions to the conception or design of the work, revised the manuscript for important intellectual content, approved the final version, and agreed to be accountable for all aspects of the work.

## Conflict of Interest

The authors declare that the research was conducted in the absence of any commercial or financial relationships that could be construed as a potential conflict of interest.
